# Clinical and Psychometric Predictors of a Positive Alcohol Use Disorder Screen after Roux-en-Y Gastric Bypass: A Cross-Sectional Study

**DOI:** 10.1007/s11695-026-08855-3

**Published:** 2026-07-30

**Authors:** Sergio Lincoln de Matos Arruda, Rafael de Negreiros Botan, Rafael Oliveira Galvão, Mariana Fiuza Gonçalves, Mariana Silva Melendez Araújo, Daniela Sampaio Carvalho Clark, João Batista de Sousa

**Affiliations:** 1 School of Medicine, Universidade de Brasília, Brasília, Brazil; 2 Bariatric Surgery, Private Practice, Brasília, Brazil; 3 Bariatric Surgery, Clínica Barinject, Brasília, Brazil; 4 HRAN/SES-DF Bariatric Surgery Outpatient Clinic, Hospital Regional da Asa Norte, Brasília, Brazil; 5 ESCS, Escola Superior de Ciências da Saúde, Brasília, Brazil; 6 Postgraduate Program in Human Nutrition, Universidade de Brasília, Brasília, Brazil; 7 Postgraduate Program in Dentistry (PPGODT), Universidade de Brasília, Brasília, Brazil; 8 Department of Surgery - Faculty of Medicine, Universidade de Brasília, Brasília, Brazil

**Keywords:** Bariatric surgery, Gastric bypass, Alcoholism, Gamma-glutamyl transferase, Biomarkers, Mass screening

## Abstract

**Background:**

Alcohol use disorder (AUD) after bariatric surgery is increasingly recognized but remains under-detected because routine screening relies on self-report. We aimed to identify clinical and psychometric predictors of a positive alcohol screen and to build a simple risk-stratification model; as a secondary objective, we critically evaluated whether a routinely available hepatic enzyme, temporally anchored gamma-glutamyl transferase (GGT), adds information.

**Methods:**

This was a single-center cross-sectional analysis of 130 adults who had undergone Roux-en-Y gastric bypass (RYGB) and completed the Cut-down/Annoyed/Guilty/Eye-opener (CAGE) and AUDIT questionnaires during routine follow-up at a private clinic in Brazil. Questionnaire responses were linked to an institutional clinical-laboratory database. The primary outcome was a positive AUDIT screen (score ≥ 8). Multivariable logistic regression with 5,000 bootstrap resamples identified independent predictors. As a secondary, exploratory analysis, the GGT measurement closest to the questionnaire date was selected within ± 6-, ± 12-, and ± 24-month windows and compared between groups, with Benjamini-Hochberg correction across a four-marker hepatic-enzyme set; a linear mixed model examined longitudinal GGT trajectories.

**Results:**

A positive AUDIT screen was identified in 32 patients (24.6%). Median age was 47 years (interquartile range [IQR] 37–58; mean 47.4 ± 12.6), and 80% were women. Male sex (OR 5.3, 95% CI 1.9–14.3) and paternal alcohol use (OR 5.5, 95% CI 2.2–13.7) were independent predictors; a two-variable model served as a candidate risk-stratification approach with selection-aware optimism-corrected internal performance (c-index 0.71; calibration slope 0.97), stratifying predicted risk from 9% (neither factor) to 75% (both, small stratum), and would require external validation before clinical use. In the AUDIT-anchored ± 6-month subgroup (*n* = 58), GGT was higher among screen-positive patients (median 31 vs. 15.5 U/L; AUC 0.72) but did not survive false-discovery-rate (FDR) correction (q = 0.055), and both medians remained within the reference range. A secondary, exploratory database analysis (Supplement) was directionally consistent at the group level but rested on an imperfect, construct-distinct label and is not confirmatory. The mixed model did not detect a divergent longitudinal GGT trajectory (β = 0.02 log-units/year, 95% CI − 0.02 to 0.06).

**Conclusions:**

After bariatric surgery, male sex and paternal alcohol use are strong, easily ascertained predictors of a positive alcohol screen in this cohort and can be translated into a simple, bedside risk-stratification approach (a candidate requiring external validation). GGT shows a weak, group-level association with alcohol use — specific within the hepatic-enzyme panel tested, yet within the reference range and uninformative at the individual level — so it may complement, but cannot replace, psychometric screening. These findings favor psychometric screening focused on identifiable risk strata, with routine GGT as at most a supportive signal.

**Supplementary Information:**

The online version contains supplementary material available at https://doi.org/10.1007/s11695-026-08855-3.

## Key Points

In 130 post-bariatric patients screened with the Alcohol Use Disorders Identification Test (AUDIT), 24.6% screened positive for problematic alcohol use (AUDIT ≥ 8). Male sex (odds ratio [OR] 5.3) and paternal alcohol use (OR 5.5) were strong independent predictors in this cohort; a simple two-variable model served as a candidate risk-stratification approach with selection-aware optimism-corrected internal performance (c-index 0.71, calibration slope 0.97), stratifying screen-positive risk from 9% (neither factor) to 75% (both), and requires external validation before clinical use.

Gamma-glutamyl transferase (GGT) carried only a weak, group-level signal of alcohol use. In the small AUDIT-anchored subgroup it did not survive correction (q = 0.055); a secondary database analysis (Supplement) was directionally consistent but exploratory and construct-distinct. Most affected patients had a normal GGT, so GGT cannot replace psychometric screening.

A secondary longitudinal mixed model with random slopes did not detect divergent GGT trajectories between groups, although statistical power for this analysis was limited.

Practice implication: psychometric screening focused on identifiable risk strata (male sex, family history) is more useful than reliance on routine liver enzymes for detecting problematic alcohol use after RYGB.

**Supplementary Information:**

The online version contains supplementary material available at https://doi.org/10.1007/s11695-026-08855-3.

## Introduction

Bariatric surgery is the most effective intervention for severe obesity, but a subset of patients develops new-onset or escalating problematic alcohol use after surgery, particularly after Roux-en-Y gastric bypass (RYGB) [1, 2]. Several mechanisms have been proposed: pharmacokinetic changes after RYGB anatomy — accelerated absorption, reduced first-pass metabolism, and higher peak ethanol concentrations [3–5] — together with psychosocial mechanisms, including cross-addiction (the emergence of a new addictive behavior after restriction of food intake), dietary restriction, and body-image stress [3]. The Longitudinal Assessment of Bariatric Surgery (LABS) cohort and subsequent studies have documented incident AUD prevalence of approximately 8% to 21% within seven postoperative years, with risk often emerging after the second year [6, 7].

Detecting post-bariatric AUD is difficult. The Alcohol Use Disorders Identification Test (AUDIT) and the Cut-down/Annoyed/Guilty/Eye-opener (CAGE) questionnaire are widely used screeners, but they rely on truthful self-report, and under-reporting is well documented in surgical populations under perceived clinical scrutiny [8, 9]. The AUDIT was developed by the World Health Organization for primary-care screening [10] and has been validated in Brazilian Portuguese [11], as has the CAGE [12]. Highly specific biomarkers of recent alcohol exposure — ethyl glucuronide, phosphatidylethanol, and carbohydrate-deficient transferrin — are not part of routine bariatric follow-up [13]. By contrast, routinely available hepatic enzymes are inexpensive and universally measured. Among them, GGT is the most responsive to low-to-moderate recent alcohol intake, whereas aspartate and alanine aminotransferase (AST, ALT) and the AST/ALT ratio rise mainly with established hepatocellular injury [14, 15]. For this reason we focused our biomarker hypothesis on GGT, while still testing the other hepatic enzymes as a family. How these enzymes behave in relation to alcohol screening after bariatric surgery, when measured close in time to the questionnaire, has not been systematically described.

We therefore had two objectives. The primary objective was to identify clinical and psychometric predictors of a positive AUDIT screen and to assemble them into a simple candidate risk-stratification model with optimism-corrected internal performance. As a secondary objective, we critically evaluated whether a routinely available hepatic enzyme adds information: we examined whether temporally anchored GGT — the measurement closest to the questionnaire date — was associated with screen-positive status, whether the association weakened as the measurement moved further from the questionnaire, and whether it withstood adjustment for metabolic confounders. We additionally examined, as a planned secondary analysis, whether longitudinal GGT trajectories diverged between groups once individual heterogeneity was accounted for, recognizing that prior work has often assumed cumulative hepatic change over time [16]. In short, we asked what is useful and what is not for detecting problematic alcohol use after RYGB, and sought to translate the answer into a practical, bedside screening aid — using a temporally anchored design that is transferable to other settings linking cross-sectional questionnaires with longitudinal laboratory data.

## Methods

### Study Design and Ethical Approval

This was a single-center cross-sectional analysis with retrospective linkage to a clinical and laboratory database. The protocol was approved by the institutional Research Ethics Committee (Comitê de Ética em Pesquisa) under protocol number CAAE 34717119.4.0000.5553. The study followed the Declaration of Helsinki and the STROBE reporting guideline for cross-sectional studies. Informed consent was waived given the retrospective use of secondary data. The de-identified dataset and analysis code are publicly available (Open Science Framework and GitHub; see Data availability).

### Population and Patient Selection

Eligible patients were adults (≥ 18 years) who had undergone RYGB at least 24 months earlier and who attended postoperative follow-up at a private bariatric clinic in Brazil. Between 2023 and 2024, 139 patients completed the CAGE and AUDIT questionnaires. These respondents were matched to the institutional clinical-laboratory database (1,869 patients; ~11,000 outpatient laboratory examinations) to retrieve their clinical history and laboratory results.

Record matching used patient name in two steps: an exact match first, followed by an approximate (“fuzzy”) string match for residual cases, with manual verification of every approximate match. Approximate matching used the RapidFuzz library (a standard text-similarity tool) requiring high similarity of first and last names plus at least two additional matching name tokens. Of the 139 respondents, 130 (93.5%) were successfully matched; the 9 unmatched patients had been referred from external services and had no institutional records, and were excluded to preserve data integrity. The final analytic sample was therefore 130 patients (Figure S3, study flow diagram). One name corresponded to two distinct homonymous respondents who shared a single laboratory identifier; both were retained for the psychometric analyses, but their ambiguous laboratory record was excluded from the biomarker analyses (see Sensitivity analyses). After this linkage error was identified, the entire name-linkage and laboratory extraction was re-audited systematically; no further ambiguous identifier was found, and the publicly deposited dataset was corrected so that it reproduces every reported number.

We did not apply an exclusion criterion for pre-existing advanced liver disease (e.g., cirrhosis or advanced fibrosis); no patient carried such a diagnosis in the clinical database, and hepatic steatosis status before surgery was recorded and used as a covariate.

### Surgical Procedure

All patients had undergone RYGB at the same service. The cohort was therefore procedurally homogeneous, which removes confounding by procedure type but precludes any comparison across procedures (e.g., sleeve gastrectomy or one-anastomosis gastric bypass).

### Psychometric Assessment

Patients self-completed the Brazilian Portuguese versions of the AUDIT (10 items) [10, 11] and CAGE (4 items) [12] during routine follow-up consultations. The primary outcome was a positive AUDIT screen, defined as a total score ≥ 8 (the conventional threshold combining hazardous use, harmful use, and probable dependence) [10]. A positive CAGE screen (≥ 1) was a secondary outcome. Additional sensitivity definitions included the continuous AUDIT score, the AUDIT-C consumption subscale (computed from the two available consumption items, because the third consumption item was corrupted in the source spreadsheet), and sex-specific thresholds (AUDIT ≥ 4 for women, ≥ 8 for men).

### Family History and Consumption Variables

Family history of alcohol use was assessed by two self-report items asking whether the patient’s father and mother, respectively, drank alcohol regularly; both paternal and maternal items were analyzed. “Regularly” was not quantified on the instrument and reflects the respondent’s own judgment; we therefore treat these items as subjective indicators of family history rather than as quantified exposure, and we note this in the Limitations. Variables that are definitionally part of the AUDIT score — current and past drinking, beverage type, and self-reported harms — were treated as descriptors and excluded from the predictor models to avoid circularity.

### Clinical and Laboratory Data

For each matched patient we retrieved all routine postoperative laboratory results, including GGT, AST, ALT, mean corpuscular volume (MCV), glycated hemoglobin (HbA1c), and a standard metabolic panel. Pre-operative comorbidities (type 2 diabetes, hypertension, dyslipidemia, hepatic steatosis, depression) were extracted from the clinical database. Concomitant hepatotoxic medications (e.g., statins, paracetamol) were not available for adjustment.

### Temporally Anchored Cross-sectional Analysis

Because the questionnaire was completed at a single time point while laboratory data spanned years, we paired each questionnaire with the laboratory measurement closest to it in time. For each patient and each predefined window (± 6, ± 12, ±24 months around the questionnaire date), we selected the single laboratory visit with the smallest time gap to the questionnaire and read all hepatic enzymes from that same visit (one value per patient per window). The ± 6-month window was the primary window; the wider windows were used to test whether the association weakened as measurements moved further from the questionnaire. The hepatic-enzyme set (GGT, AST, ALT, AST/ALT ratio) was compared between groups with Benjamini-Hochberg false-discovery-rate (FDR) correction; MCV, a hematologic marker, was reported separately (Supplementary Table S1). As a sensitivity check, an alternative anchoring that instead selected the closest GGT-bearing measurement per patient yielded a slightly smaller GGT FDR q value (≈ 0.04 versus 0.055); because both lie immediately around the 0.05 threshold, the interpretation of GGT as a borderline, non-robust signal is unchanged by the anchoring rule.

### Statistical Analysis

Continuous variables are summarized as median (IQR) and, to aid interpretation, also as mean ± standard deviation (SD) for the main descriptors; categorical variables as n (%). Groups were compared with the Mann-Whitney U or Fisher exact test. The primary model was a parsimonious multivariable logistic regression of AUDIT-positive status on the two predictors that were robust across model specifications — male sex and paternal alcohol use (32 events; EPV = 16); age and time since surgery were retained in a sensitivity model and were not significant. Confidence intervals were obtained from 5,000 bootstrap resamples, and variance inflation factors were examined. Although male sex and paternal alcohol use are well-established a priori risk factors for alcohol misuse, the risk model was not formally pre-registered; to avoid overstating its performance we therefore treat it as a candidate risk-stratification approach requiring external validation and estimated its internal performance with a conservative, selection-aware optimism correction: rather than correcting a fixed model, we re-ran the entire variable-selection procedure (backward elimination at *p* < 0.05 from a non-circular candidate set of male sex, paternal and maternal alcohol use, age, time since surgery, current BMI, and education) inside each of 1,000 bootstrap resamples, so that any optimism attributable to data-driven selection is incorporated into the corrected c-index (Harrell-Steyerberg method). We also recorded how often each candidate was selected across resamples as a stability check. Calibration was summarized by the bootstrap calibration slope and the Brier score, and predicted risks were tabulated by predictor combination as a simple bedside aid. We disclose that the reported c-index reflects this selection-aware correction; the apparent (uncorrected) value is reported alongside for transparency. Because the same data informed both the choice of predictors and their estimation, the odds ratios and confidence intervals for the final model are descriptive summaries that may be optimistic (anti-conservative) and, like the model’s discrimination, require external validation. For the secondary GGT analysis, discrimination was summarized with the AUC and a bootstrap 95% CI, the continuous AUDIT score was correlated with GGT (Spearman) as a dose-response check, and GGT was related to sex and current BMI to assess metabolic confounding. Additional methodological detail (mixed-model specification, likelihood-ratio tests, leave-one-out diagnostics) is provided in the Supplementary Methods. Analyses used Python 3.13 (pandas, statsmodels, scikit-learn, scipy).

We report the longitudinal slope × screen interaction regardless of significance. The anchored cross-sectional GGT analysis was exploratory and not pre-registered; we therefore applied FDR correction within the hepatic-enzyme set and report the GGT result as hypothesis-generating. The full analysis is documented in a publicly deposited, post-hoc analytical protocol and reproducible code (see Data availability).

### Secondary Large-Scale Database Analysis (Supplement)

As a secondary, exploratory analysis conducted during revision in response to the reviewers’ concern about the small AUDIT-anchored subgroup, we examined the GGT–alcohol association in the broader source cohort using an independent, non-psychometric label derived from the pre-operative clinical history; because that label is a different construct from the post-operative AUDIT and is imperfect, this analysis is reported in full in the Supplementary Material (Supplementary Methods S2, Supplementary Results, Figure S4, Supplementary Table S5) and is summarized in a single sentence in the main Results.

### Sensitivity Analyses

Because one laboratory identifier was shared by two homonymous respondents, we examined three handling rules for the ± 6-month window: excluding the ambiguous record (primary), and assigning it to either screen group. We also repeated the primary model excluding both homonyms. Multiplicity, model stability, and the limited screen-positive subgroup are addressed quantitatively in the Discussion and Supplement.

## Results

### Sample Characteristics

Of 130 patients (Table 1), 80% were women and the median age was 47 years (IQR 37–58; mean 47.4 ± 12.6). The median time since surgery was 92.5 months (IQR 45–151), pre-operative body mass index (BMI) was 39.7 kg/m² (IQR 37.1–42.7; mean 40.2 ± 4.4), and current BMI was 28.4 kg/m² (IQR 25.8–31.9; mean 29.3 ± 4.8). A positive AUDIT screen (≥ 8) was identified in 32 patients (24.6%): 20 with hazardous use, 6 with harmful use, and 6 with probable dependence. A positive CAGE screen (≥ 1) occurred in 33 patients (25.4%). Paternal and maternal alcohol use were reported by 42 (32.3%) and 20 (15.4%) patients, respectively.

**Table 1:** Sample characteristics (*N* = 130)

Characteristic	Value — median (IQR); mean ± SD, or *n* (%)
Age, years	46.5 (37.0–57.8); 47.2 ± 12.5
Female sex	104 (80.0%)
Time since surgery, months	92.5 (45.0–151.8); 99.6 ± 59.4
Pre-operative BMI, kg/m²	39.7 (37.1–42.9); 40.7 ± 5.0
Current BMI, kg/m²	28.4 (25.8–31.9); 29.3 ± 5.1
AUDIT-positive (≥ 8)	32 (24.6%)
CAGE-positive (≥ 1)	33 (25.4%)
Paternal alcohol use	42 (32.3%)
Maternal alcohol use	20 (15.4%)

*AUDIT*, Alcohol Use Disorders Identification Test; *BMI*, body mass index; *IQR*, interquartile range; *SD*, standard deviation

### Independent Predictors and a Simple Risk Model (Primary Analysis)

In the parsimonious model (Table 2), male sex (OR 5.3, 95% CI 1.9–14.3) and paternal alcohol use (OR 5.5, 95% CI 2.2–13.7) were independent predictors of a positive AUDIT screen (EPV = 16; all variance inflation factors < 2.0). In bootstrap resampling, both were selected as significant in ≥ 90% of resamples. Adding age and time since surgery did not improve the model and neither was significant (OR 0.97/year for each). Univariate associations with younger age, shorter time since surgery, lower religiosity, and lower education did not survive adjustment. A positive CAGE screen showed weaker associations overall, consistent with its reliance on lifetime perception and its previously described limitations for new-onset phenotypes [17, 18].

**Table 2:** Multivariable predictors of a positive AUDIT screen and internal validation

Predictor	OR (95% CI)	*p*
Male sex	4.87 (1.78–13.32)	0.0021
Paternal alcohol use	4.91 (1.92–12.54)	0.0009
Age, per year (sensitivity)	0.97 (0.93–1.01)	0.1832
Time since surgery, per year (sensitivity)	0.97 (0.87–1.08)	0.57

Primary model: male sex + paternal alcohol use (32 events; EPV = 16). Internal validation: apparent c-index 0.75; selection-aware optimism-corrected c-index 0.71 (variable selection re-run inside each bootstrap; both predictors reselected in ~86% of resamples); calibration slope 0.97; Brier 0.14. Selection was data-driven, not pre-specified; the model is a candidate tool requiring external validation. *OR*, odds ratio; *CI*, confidence interval; *EPV*, events per variable

On internal assessment with a selection-aware optimism correction, the two-variable model showed moderate discrimination and good internal calibration: apparent c-index 0.75; selection-aware optimism-corrected c-index 0.71 (bootstrap optimism ≈ 0.04 — substantially larger than the ≈ 0.01 obtained when only a fixed model is corrected, precisely because it now also accounts for data-driven variable selection); internal calibration slope 0.97; and Brier score 0.14. Across the 1,000 resamples, the backward procedure reselected male sex and paternal alcohol use in 86% of samples each, whereas no other candidate was selected in more than one-third, confirming the stability of the two-variable model. Predicted risk of a positive screen rose from 9% in patients with neither factor, to 36–37% with one factor, to 75% in men with paternal alcohol use (observed 11/12, a small stratum with correspondingly wide uncertainty); the full risk table is shown in Table 3. As an apparent, descriptive summary of these pre-specified strata (small per-stratum numbers, no threshold optimisation), the presence of either factor corresponded to an apparent sensitivity of 72% and negative predictive value of 88% for a positive screen, while the presence of both factors corresponded to an apparent positive predictive value of 92% and specificity of 99% with low sensitivity (34%; *n* = 12). These are apparent (internal) descriptive figures, not a validated decision rule, and require external validation before any clinical use. These two bedside variables therefore provide a candidate risk-stratification approach that, while internally well-calibrated, requires external validation before clinical use.

**Table 3:** Predicted risk of a positive screen by predictor combination

Male sex	Paternal alcohol use	Predicted risk	Observed
No	No	9%	9/74 (12%)
No	Yes	37%	9/30 (30%)
Yes	No	36%	3/14 (21%)
Yes	Yes	75%	11/12 (92%)

### Comparison of Included Versus Excluded Patients

To assess potential selection bias arising from the reduction to the laboratory subgroup, we compared patients who contributed a paired ± 6-month GGT measurement (*n* = 67) with those who did not (*n* = 63) (Supplementary Table S4). Groups were similar in sex, age, current BMI, time since surgery, paternal and maternal alcohol use, and screen-positive rate; all standardized mean differences were small (maximum 0.27, for weight regain). This argues against major selection bias on measured characteristics, although unmeasured differences cannot be excluded.

### Anchored GGT as a Concurrent Correlate (Secondary, Exploratory)

**Fig. 1:**
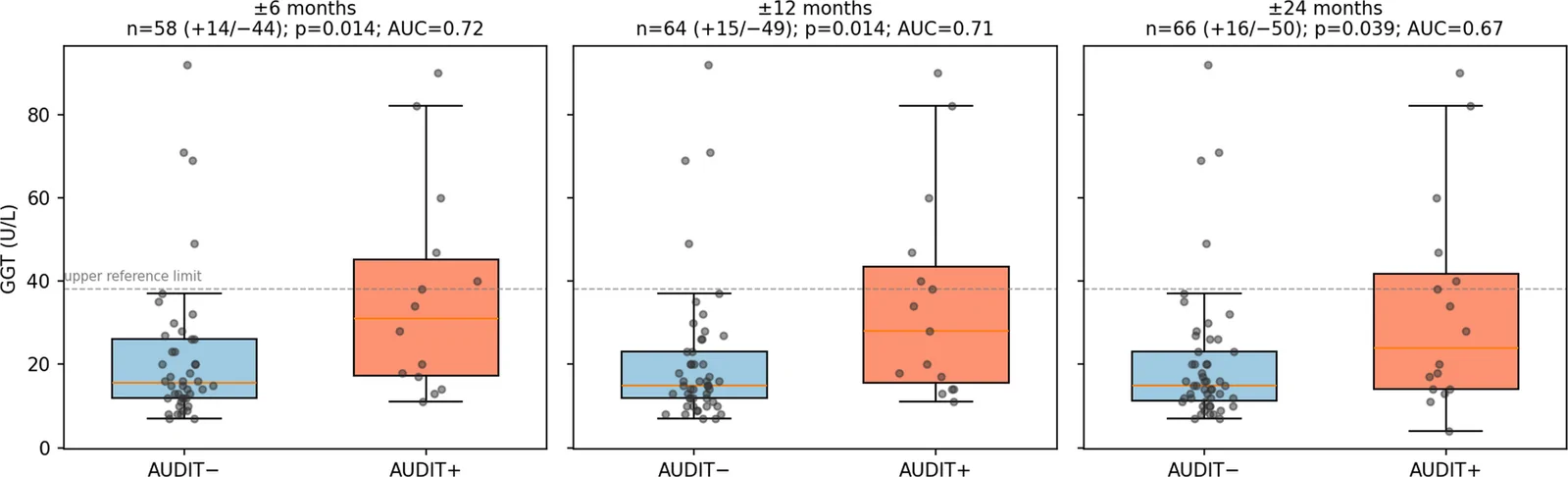
Anchored GGT by AUDIT screening status across temporal windows (A: ±6 months; B: ±12 months; C: ±24 months). Boxplots show the GGT measurement closest to the questionnaire. Mann-Whitney p values and AUC are shown above each panel

**Fig. 2:**
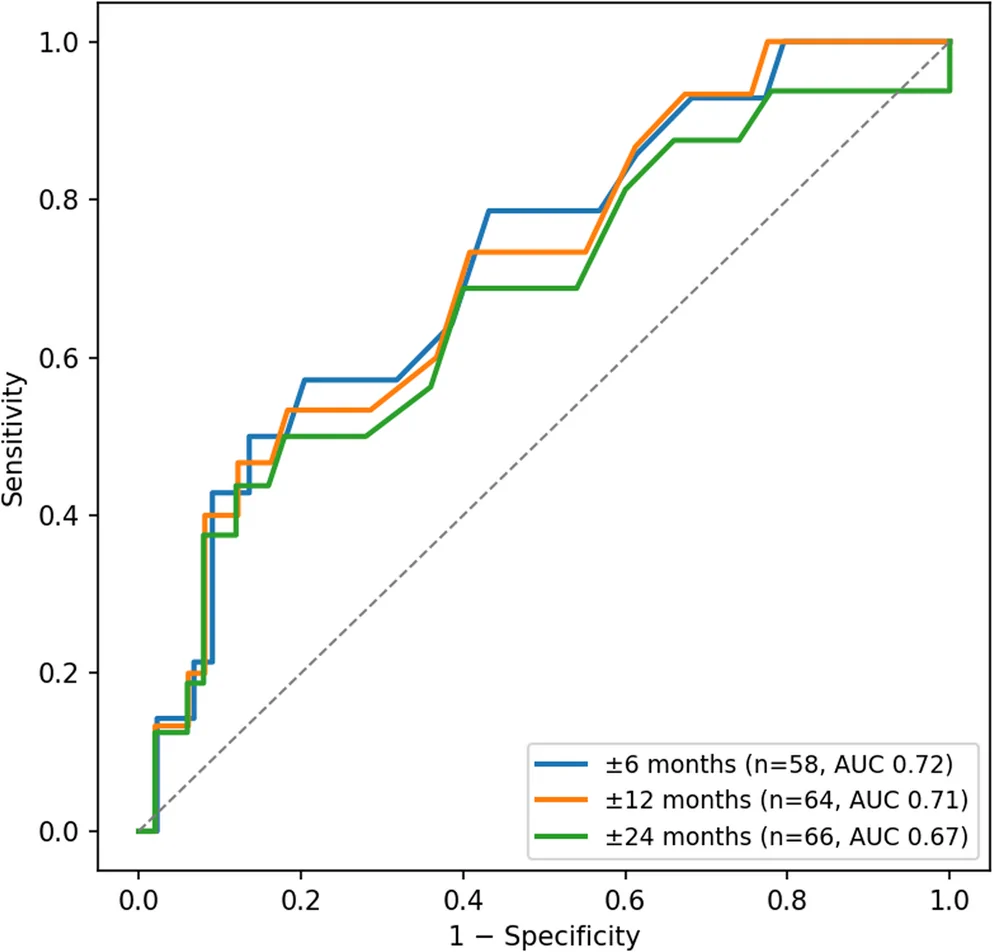
ROC curves for anchored GGT discriminating a positive AUDIT screen across windows (± 6 m, *n* = 58, AUC 0.72; ±12 m, *n* = 64, AUC 0.71; ±24 m, *n* = 66, AUC 0.67)

In the ± 6-month window (*n* = 58 patients with a paired GGT), GGT was higher in screen-positive than screen-negative patients (median 31 vs. 15.5 U/L; mean 38 ± 24 vs. 22 ± 17; *p* = 0.014; AUC 0.72, 95% CI 0.56–0.87; Fig. 1A). Discrimination attenuated as the window widened to ± 12 months (AUC 0.71, *n* = 64) and ± 24 months (AUC 0.67, *n* = 66) (Figs. 1B–C and 2). However, several findings temper this signal. Within the hepatic-enzyme set, GGT had the smallest p value but did not survive FDR correction (q = 0.055 in the ± 6-month window); AST, ALT, and AST/ALT ratio were not associated (Table 4). GGT did not correlate with the continuous AUDIT score (Spearman ρ = 0.16, *p* = 0.23), so a dose-response relationship was not evident. GGT was instead associated with current BMI (ρ = 0.28, *p* = 0.03) and was numerically higher in men (median 27 vs. 17 U/L), indicating that adiposity and sex — both linked to GGT independently of alcohol — confound the association. MCV was not associated in any window (Supplementary Table S1). Both group medians remained within the laboratory reference range.

**Table 4:** Hepatic-enzyme set in the ±6-month window (*n* = 58), with FDR correction

Marker	*p* value	q value (BH)	Survives q < 0.05
GGT	0.0137	0.0549	No
AST	0.971	0.971	No
ALT	0.4342	0.579	No
AST/ALT ratio	0.2165	0.433	No

Benjamini-Hochberg (BH) false-discovery-rate correction across the four-marker set. GGT had the smallest *p* value but did not survive correction. *AST*, aspartate aminotransferase; *ALT*, alanine aminotransferase; *GGT*, gamma-glutamyl transferase

### Secondary Large-Scale Database Analysis (Summary; Full Detail in Supplement)

In the secondary database analysis (Supplementary Results, Figure S4), higher GGT was directionally associated with a pre-operative, trait-level documented-misuse label after adjustment (*n* = 1,322 with GGT; adjusted OR per log-GGT 2.4, 95% CI 1.4–4.0), specifically for GGT and not AST/ALT; however, because the label is exploratory, imperfect (positive predictive value 50% against the AUDIT), and a different construct from the post-operative screen, and because most labelled patients had a GGT within the reference range, we treat this only as convergent, hypothesis-level evidence of a weak group-level signal that bounds rather than rescues the biomarker claim and does not support individual GGT screening.

In sensitivity analyses for the shared laboratory identifier, the ± 6-month AUC ranged from 0.68 to 0.73 depending on how the ambiguous record was handled, and the direction of the association was unchanged. Excluding both homonyms from the primary model left the predictor estimates essentially unchanged.

### Longitudinal GGT Trajectories (Secondary)

Using all postoperative GGT measurements (*n* = 379 measurements, 78 patients), a linear mixed model with random intercept and random slope per patient did not detect a divergent trajectory between groups (slope × screen interaction β = 0.02 log-units/year, 95% CI − 0.02 to 0.06, *p* = 0.26; Figure S1, Supplementary Table S2). The random-slope structure was statistically necessary (likelihood-ratio test *p* < 0.001), indicating substantial individual heterogeneity in trajectories. Sensitivity analyses using the continuous AUDIT score, AUDIT-C, and sex-specific thresholds were similarly null, and adjustment for steatosis, diabetes, dyslipidemia, and hypertension did not change the result. We note that statistical power for detecting a between-group slope difference was limited.

## Discussion

In a Brazilian post-RYGB cohort, this study asked a deliberately practical question: what is useful, and what is not, for detecting problematic alcohol use after surgery. Our contribution is pragmatic and methodological rather than a claim of new risk factors. Clinically, we translate two well-established but under-used risk factors — male sex and paternal alcohol use, both strong independent predictors here — into a simple, bariatric-specific risk-stratification aid: a two-variable model with acceptable, selection-aware optimism-corrected internal performance (c-index 0.71, well calibrated) that stratified screen-positive risk from 9% to 75% from information obtainable at the bedside in seconds. The value is not that these predictors are novel, but that they remain informative after surgery and combine into an actionable risk gradient; the model is a candidate that still requires external validation before clinical use. Methodologically, we introduce a temporally anchored design — pairing each questionnaire with the laboratory measurement closest in time — that is transferable to any study linking cross-sectional instruments with longitudinal laboratory data, and we use it to delineate precisely what a routine biomarker can and cannot contribute. The more nuanced part is the routine hepatic enzyme itself. In the small AUDIT-anchored subgroup, GGT was higher in screen-positive patients but did not survive multiplicity correction and stayed within the reference range. A secondary, exploratory database analysis (Supplement) was directionally consistent at the group level — the association survived adjustment and was specific to GGT — but it rested on an imperfect, construct-distinct label, and most labelled patients still had a normal GGT. The coherent reading is that GGT carries only a weak, group-level signal of alcohol use, insufficient for individual screening: a routinely available test that may support, but cannot replace, psychometric screening.

The strong and consistent association of male sex (OR ≈ 5) and paternal alcohol use (OR ≈ 5) is consistent with the LABS cohort and other reports of male predominance in post-bariatric AUD [6, 17] and with family history as an established, transdiagnostic risk factor for substance-use disorders in the general population [19]. Importantly, these are well-recognized risk factors for alcohol misuse outside the bariatric setting; our data indicate that they remain informative after surgery rather than representing a bariatric-specific mechanism. Because both are simple to ascertain at the bedside, they may help focus screening on higher-risk patients. We do not interpret the coefficients as causal.

The temporal-anchoring design is a methodological feature worth underscoring, but its yield here is modest and instructive. By selecting the laboratory measurement closest to each questionnaire and varying the window, we found that the GGT signal was strongest at the shortest interval (± 6 months) and weakened with wider windows — a pattern compatible with GGT’s responsiveness to recent intake [14, 15, 20]. However, several caveats are decisive. The screen-positive subgroup was small (14 patients in the primary window), the AUC confidence interval was wide, and the association did not survive FDR correction (q = 0.055). There was no dose-response with the continuous AUDIT score. GGT also correlated with current BMI and was higher in men, both of which can raise GGT independently of alcohol; however, the secondary database analysis (Supplement) suggested confounding was not the whole story, since the association survived adjustment for BMI, steatosis, and diabetes and was specific to GGT rather than AST/ALT, though that analysis is itself exploratory and construct-distinct. The limiting fact is clinical rather than statistical: although the median difference was roughly two-fold, both medians lay within the reference range and most patients with documented misuse had a normal GGT, so the enzyme is too insensitive to serve as a stand-alone test in individual patients even though a weak group-level association is present. Within the hepatic-enzyme set, AST, ALT, and the AST/ALT ratio were not associated, in keeping with their lower sensitivity to low-to-moderate intake [14, 15].

Our longitudinal model did not detect divergent GGT trajectories between groups. We interpret this cautiously: it indicates the absence of a detectable cumulative divergence in these data, not proof that no cumulative process exists, because power for a between-group slope difference was limited and follow-up was irregular. This contrasts with work that has inferred progressive hepatic change in long-term post-bariatric patients using simpler models [16]; allowing patient-specific slopes, as the data require, removes the spurious group-level signal that a random-intercept-only model can generate. Taken together with the cross-sectional gradient, the most parsimonious reading is that any GGT signal in this cohort is contemporaneous rather than cumulative — a hypothesis for prospective testing rather than an established conclusion. Looking forward, the temporal-anchoring approach would be most informative if applied prospectively to a larger, multicenter sample with a validated alcohol-use outcome and a specific alcohol biomarker (e.g., phosphatidylethanol); the present secondary database analysis, despite its imperfect label, can serve as a hypothesis-generating pilot for such a study and for estimating the sample size needed to detect any incremental screening value of routine GGT.

Several unmeasured factors may influence GGT independently of alcohol and could not be accounted for, including smoking, hepatic steatosis severity and fibrosis (only binary pre-operative steatosis was available), nonalcoholic fatty liver disease progression, weight regain, nutritional status, and hepatotoxic medications. Any of these could contribute to the observed association; their absence is an important limitation of the biomarker analysis. We were also unable to compare alcohol-related outcomes across surgical procedures, because the cohort was uniformly RYGB; while this removes procedure confounding, it prevents extrapolation to sleeve gastrectomy or one-anastomosis gastric bypass, for which the pharmacokinetic and behavioral risks may differ [4, 5].

### Limitations

This study has several limitations. First, it is a single-center, retrospective analysis at a private bariatric clinic in Brazil, which limits generalizability to other settings and health systems. Second, AUDIT and CAGE are screening instruments, not diagnostic tools; we cannot infer DSM-5 alcohol use disorder. Third, the biomarker findings rest on a small subgroup — only 14 screen-positive patients in the primary ± 6-month window — so ROC and AUC estimates are imprecise, multiplicity is a concern, and subgroup results are not robust; we addressed this with FDR correction, bootstrap resampling, and sensitivity analyses, but residual type I error cannot be excluded. Fourth, the anchored association is correlational and does not establish direction or causation. Fifth, important confounders of GGT — smoking, fibrosis/steatosis severity, weight regain, nutritional status, and hepatotoxic medications — and the more specific alcohol biomarkers (phosphatidylethanol, carbohydrate-deficient transferrin) were not available. Sixth, family-history items were not quantified. Seventh, the reduction from 139 respondents to the laboratory subgroup raises the possibility of selection bias, although included and excluded patients were similar on measured characteristics (Supplementary Table S4). Eighth, the secondary large-scale database analysis (reported in the Supplement, not in the main results) relied on a text-derived label of documented alcohol use rather than a standardized instrument. Unlike the AUDIT, free-text documentation is heterogeneous across clinicians, and the absence of a note does not reliably mean abstinence — it may mean the question was not asked or not recorded. Such non-differential misclassification of exposure generally biases associations toward the null, so it is more likely to have attenuated than inflated the observed effect; differential misclassification (a note prompted by an abnormal GGT) is argued against on three grounds: the alcohol history was recorded pre-operatively and therefore before the post-operative GGT values analysed, making reverse documentation temporally implausible; 69% of patients with documented misuse had a normal GGT; and the association was specific to GGT over AST/ALT. We are deliberate about the two edges of this temporal argument: recording the label before surgery removes reverse documentation by the post-operative GGT, but it also means the label is a pre-operative, trait-level construct that is not the same as the post-operative AUDIT screen, and it does not by itself exclude confounding-by-indication or differential clinical surveillance of patients flagged as higher-risk. The label is therefore imperfect (positive predictive value 50% against AUDIT), trait-level rather than time-anchored, and a different construct from the outcome. For these reasons the large-scale analysis is presented strictly as convergent evidence on a weak group-level GGT–alcohol signal, not as a stand-alone, time-anchored, or confirmatory result, and not as evidence that GGT detects post-operative alcohol use disorder in individuals. Finally, self-report is subject to under-reporting, which may bias associations toward the null.

## Conclusions

After bariatric surgery, male sex and paternal alcohol use were strong, easily ascertained predictors of a positive alcohol screen in this cohort and may help focus surveillance; combined into a two-variable model they provide a candidate risk-stratification approach whose good internal, optimism-corrected performance still requires external validation before clinical use. Temporally anchored GGT showed a modest, borderline association that was strongest when measured close to the questionnaire and remained within the reference range; this exploratory signal is best regarded as hypothesis-generating and requires confirmation in larger, prospective, multicenter studies before any clinical use. These findings support pairing psychometric screening with attention to identifiable risk strata in routine bariatric follow-up.

## Supplementary Information

Below is the link to the electronic supplementary material.

Supplementary Material 1

Supplementary Material 2

## Data Availability

De-identified analytical dataset and reproducible analysis code are publicly deposited in an institutional reproducibility repository under a permanent DOI. The DOI and repository URL are provided in the Title Page File and will be made available to readers upon acceptance. The public dataset replaces patient names with sequential study identifiers (P001–P130) generated locally and irreversibly; direct identifiers (national ID, hospital ID, full birthdate) are not included.
