# Supplementary material for: Clinical and Psychometric Predictors of a Positive Alcohol Use Disorder Screen after Roux-en-Y Gastric Bypass: A Cross-Sectional Study
Source: Obes Surg. 2026 Jul 30;36(9):4677–86. doi: 10.1007/s11695-026-08855-3 (PMC13562264; doi:10.1007/s11695-026-08855-3)
Supplement: Supplementary file 1 [file 11695_2026_8855_MOESM1_ESM.docx]

# STROBE Statement — checklist for cross-sectional studies

*Manuscript: "Clinical and psychometric predictors of a positive alcohol use disorder screen after Roux-en-Y gastric bypass: a cross-sectional study." Section/location references are to the revised (clean) manuscript.*

| Item | # | Recommendation — how addressed | Location |
| --- | --- | --- | --- |
| Title and abstract | 1 | (a) Cross-sectional design indicated in the title ("a cross-sectional study"). (b) Structured abstract with Background/Methods/Results/Conclusions. | Title; Abstract |
| **Introduction** |  |  |  |
| Background/rationale | 2 | Rationale for predictors of a positive alcohol screen after RYGB and for evaluating GGT. | Introduction |
| Objectives | 3 | Two objectives stated: (i) predictors + candidate risk model; (ii) critical evaluation of GGT. | Introduction (final paragraph) |
| **Methods** |  |  |  |
| Study design | 4 | Single-center cross-sectional analysis with retrospective laboratory linkage. | Methods — Study design |
| Setting | 5 | Private bariatric clinic in Brazil; questionnaires 2023–2024; up to multi-year laboratory follow-up. | Methods — Population |
| Participants | 6 | Adults ≥18 y, ≥24 months post-RYGB, who completed CAGE/AUDIT; linkage by name (exact then verified fuzzy). | Methods — Population and patient selection |
| Variables | 7 | Outcome: positive AUDIT (≥8). Predictors: male sex, paternal alcohol use (and candidates). GGT and hepatic enzymes; covariates. | Methods — Variables; Statistical analysis |
| Data sources/measurement | 8 | Self-completed Brazilian-Portuguese AUDIT/CAGE; laboratory results from institutional database; temporally anchored GGT. | Methods — Psychometric assessment; Anchored analysis |
| Bias | 9 | Selection bias addressed (included vs excluded, SMD ≤0.27); homonym linkage error corrected and re-audited; non-differential misclassification discussed. | Methods; Results; Limitations |
| Study size | 10 | 139 respondents → 130 analysed; anchored subgroups (±6 m n=58, etc.); flow diagram. | Methods; Figure S3 |
| Quantitative variables | 11 | Continuous as median (IQR) and mean ± SD; categorical as n (%). | Methods — Statistical analysis; Table 1 |
| Statistical methods | 12 | Multivariable logistic regression; 5,000 bootstrap CIs; selection-aware optimism-corrected internal validation; FDR (Benjamini-Hochberg); mixed model; sensitivity analyses. | Methods — Statistical analysis; Supplementary Methods |
| **Results** |  |  |  |
| Participants | 13 | Numbers at each stage; reasons for non-inclusion (9 external referrals); flow diagram. | Results — Sample; Figure S3 |
| Descriptive data | 14 | Demographics, anthropometrics, family history; included vs excluded comparison. | Results; Table 1; Supplementary Table S4 |
| Outcome data | 15 | AUDIT-positive 32 (24.6%); CAGE-positive 33 (25.4%). | Results — Sample characteristics |
| Main results | 16 | Adjusted OR (95% CI) for predictors; candidate risk model (c-index 0.71, calibration 0.97; risk 9%→75%); anchored GGT (q=0.055). | Results; Table 2; Table 3 |
| Other analyses | 17 | Sensitivity (homonym handling AUC 0.68–0.73), subgroup, longitudinal mixed model, and a secondary large-scale database analysis (Supplement). | Results; Supplementary Results |
| **Discussion** |  |  |  |
| Key results | 18 | Predictors and risk model are the useful, validated contribution; GGT is a weak group-level signal, not a stand-alone screen. | Discussion (first paragraph) |
| Limitations | 19 | Single-center; small biomarker subgroup; unmeasured confounders; text-label analysis imperfect/construct-distinct; no pre-registration. | Limitations |
| Interpretation | 20 | Cautious, hypothesis-generating interpretation; external validation required for the risk model. | Discussion; Conclusions |
| Generalisability | 21 | Limited to single private Brazilian center; uniform RYGB; not extrapolated to other procedures. | Limitations; Conclusions |
| **Other information** |  |  |  |
| Funding | 22 | Stated in the Statements section. | Statements |
