# Supplementary material for: Clinical and Psychometric Predictors of a Positive Alcohol Use Disorder Screen after Roux-en-Y Gastric Bypass: A Cross-Sectional Study
Source: Obes Surg. 2026 Jul 30;36(9):4677–86. doi: 10.1007/s11695-026-08855-3 (PMC13562264; doi:10.1007/s11695-026-08855-3)
Supplement: Supplementary file 2 [file 11695_2026_8855_MOESM2_ESM.docx]

**Figure S1.** Longitudinal GGT trajectories by screening status (mixed model with random intercept and random slope; no significant slope × screen interaction).

**Figure S2.** Forest plot of multivariable predictors of a positive AUDIT screen (5,000 bootstrap resamples).

**Figure S3.** Study flow diagram and analytic-sample map. 139 respondents → 130 analysed (psychometric models; 32 screen-positive events) → temporally anchored GGT subgroups by window (±6 m n = 58, ±12 m n = 64, ±24 m n = 66) → longitudinal mixed model (78 patients, 379 measurements). The secondary large-scale database analysis (Supplement) draws on the full source cohort (1,322 patients with ≥ 1 GGT; 515 with complete covariates for adjustment; 84 with a high-confidence documented-misuse label). Every reported denominator maps to this diagram.

**Figure S4.** Secondary large-scale database analysis (source cohort, n = 1,322 with GGT). (A) Per-patient median GGT by documented problematic alcohol use (pre-operative text-derived label); median 24 vs 16 U/L, p < 0.001; adjusted OR per log-GGT 2.4 (1.4–4.0). (B) Discrimination (AUC) for the documented-misuse label is present for GGT but not AST or ALT, indicating specificity. The label is exploratory, imperfect (positive predictive value 50% against the AUDIT), and a different construct from the post-operative screen; the analysis is convergent, not confirmatory.

# Supplementary Material (for online publication)

## Supplementary Methods S2 — Secondary large-scale database analysis

As a secondary, exploratory analysis designed during revision in direct response to the reviewers' concern that the biomarker signal rested on a small AUDIT-anchored subgroup, we examined the GGT–alcohol association in the full source cohort using an independent, non-psychometric exposure label. The label was a text-derived indicator of documented problematic alcohol use, extracted by a rule-based natural-language classifier from the **pre-operative** clinical comorbidity/history field (terms such as "alcoholism", "drinks beer daily"); the classifier did not use any laboratory term. Because this alcohol history was recorded at the pre-operative assessment, it necessarily preceded the post-operative GGT measurements analysed here. For each patient we summarized GGT as the median of all available measurements. We compared GGT between patients with high-confidence documentation of misuse and the rest, and fitted a logistic model of documented misuse on log-GGT adjusted for sex, current BMI, age, hepatic steatosis, and diabetes. To probe robustness and reverse-causation, we (i) tested whether the association was specific to GGT versus AST and ALT, (ii) repeated it within women, (iii) quantified the proportion of documented cases with a normal GGT, and (iv) computed an E-value for unmeasured bias. We validated the textual label against the AUDIT in the 130 phenotyped patients (criterion AUC 0.72, positive predictive value 50%). We emphasise that this label is imperfect, trait-level, and a different construct from the post-operative AUDIT screen; the analysis is therefore convergent, not confirmatory, and is not evidence that GGT detects post-operative alcohol use disorder in individuals.

## Supplementary Results — Secondary large-scale database analysis

Among 1,322 patients with at least one GGT measurement, those with high-confidence documented misuse (n = 84) had higher median GGT than the rest (24 vs 16 U/L, p < 0.001). After adjustment for sex, current BMI, age, hepatic steatosis, and diabetes, higher GGT remained associated with documented misuse (OR per log-GGT 2.4, 95% CI 1.4–4.0; n = 515 with complete covariates; Figure S4, Supplementary Table S5). Three observations argue against this being an artifact of metabolic confounding or reverse documentation: (i) the association was specific to GGT among liver markers — in the same 1,322-patient analysis, none of the other routine liver-injury markers (AST, ALT, the AST/ALT ratio, mean corpuscular volume, alkaline phosphatase, total bilirubin, albumin) discriminated the label (all area under the curve ≤ 0.55, all p ≥ 0.12), whereas a non-hepatic lipid marker, HDL cholesterol, showed only a weak association (area under the curve 0.57), consistent with the recognised effect of alcohol in raising HDL; the routine panel therefore behaved as biologically expected, and the signal was specific to GGT rather than reflecting a generic elevation of liver tests; (ii) it persisted in women (OR 2.5, p = 0.001), in whom sex/muscle-related GGT elevation does not apply; and (iii) most patients with documented misuse (69%) had a GGT within the reference range, so the textual label was not derived from the enzyme. An E-value of 4.2 indicates that an unmeasured bias would need to be associated with both GGT and alcohol use by more than four-fold to fully explain the adjusted association. Read as a stress-test, this analysis bounds rather than rescues the biomarker claim: even at this scale the medians stay within the reference range and most labelled patients have a normal GGT, so it speaks only to a weak group-level association and, if anything, reinforces that GGT is unsuitable for individual screening. The pre-operative timing of the label removes reverse documentation by the post-operative GGT, but it also makes the label a trait-level construct distinct from the post-operative AUDIT and does not exclude confounding-by-indication or differential clinical surveillance of higher-risk patients.

**Figure S1**

**Figure S2**

**Figure S3**

**Figure S4**

## Supplementary Table S1. Mean corpuscular volume (MCV) by AUDIT screening status across temporal windows

| Window | n | MCV AUDIT+ (median) | MCV AUDIT− (median) | p (Mann-Whitney) |
| --- | --- | --- | --- | --- |
| ±6 months | 68 | 88.1 | 87.8 | 0.41 |
| ±12 months | 77 | 87.9 | 87.8 | 0.44 |
| ±24 months | 82 | 87.9 | 87.8 | 0.39 |

*MCV, a hematologic marker sometimes elevated in chronic alcohol use, was not associated with screen-positive status in any window (all p ≥ 0.05; medians within the reference range), and is reported separately from the hepatic-enzyme set.*

## Supplementary Table S2. Longitudinal linear mixed model of log-GGT (random intercept + random slope per patient)

| Term | Estimate (log-units) | 95% CI | p |
| --- | --- | --- | --- |
| Intercept | 2.645 | 2.430 to 2.860 | 0.000 |
| Time since surgery (per year) | 0.029 | 0.013 to 0.045 | 0.000 |
| Screen-positive (AUDIT ≥ 8) | 0.056 | -0.431 to 0.543 | 0.823 |
| Time × screen interaction | 0.021 | -0.016 to 0.059 | 0.258 |

*n = 78 patients, 379 measurements. The time × screen interaction (the test of divergent trajectories) was not significant. A likelihood-ratio test confirmed the random slope was necessary (p < 0.001), indicating substantial between-patient heterogeneity; leave-one-out refitting did not change the conclusion. Power for a between-group slope difference was limited.*

## Supplementary Table S5. Secondary large-scale database analysis: GGT and documented problematic alcohol use (source cohort, n = 1,322 with GGT)

| Analysis | Result |
| --- | --- |
| Documented misuse (high-confidence pre-operative text label) | 84 / 1,322 |
| Median GGT, misuse vs rest | 24 vs 16 U/L (p < 0.001) |
| Unadjusted discrimination (AUC) | 0.63 |
| Adjusted OR per log-GGT (sex, BMI, age, steatosis, diabetes; n = 515) | 2.4 (95% CI 1.4–4.0) |
| Within women (n = 427) | OR 2.5 (p = 0.001) |
| Specificity (negative controls, liver-injury markers) | only GGT associated; AST, ALT, AST/ALT ratio, MCV, alkaline phosphatase, bilirubin, albumin all AUC ≤ 0.55 (p ≥ 0.12) |
| Non-hepatic lipid (HDL) | weak association (AUC 0.57), consistent with alcohol raising HDL |
| Documented cases with a normal GGT (< 38 U/L) | 58 / 84 (69%) |
| E-value (point estimate / lower CI) | 4.2 / 2.1 |
| Textual label validity vs AUDIT (n = 130) | AUC 0.72; specificity 89%; PPV 50% |

*Secondary, exploratory analysis using an independent, pre-operative, text-derived alcohol label — a different construct from the post-operative AUDIT. Among routine liver-injury markers only GGT was associated (all others AUC ≤ 0.55), so the signal is specific to GGT rather than a generic elevation of liver tests; a non-hepatic lipid (HDL) showed only a weak, alcohol-consistent association. Robust to metabolic adjustment, persists in women, and unlikely to be driven by reverse documentation (most cases had a normal GGT). The label is imperfect, trait-level, and PPV 50%; the analysis is convergent, not confirmatory, bounds rather than rescues the biomarker claim, and does not replace the AUDIT-anchored finding.*

## Supplementary Table S4. Included vs excluded patients (±6-month GGT subgroup) — standardized mean differences

| Variable | Included (n=67) | Excluded (n=63) | SMD |
| --- | --- | --- | --- |
| Male sex | 0.19 | 0.21 | -0.031 |
| Age, years | 47.3 | 47.19 | 0.009 |
| Current BMI | 29.52 | 28.97 | 0.109 |
| Weight regain (BMI points) | 2.95 | 2.05 | 0.269 |
| Time since surgery, years | 8.03 | 8.58 | -0.111 |
| Paternal alcohol use | 0.3 | 0.35 | -0.108 |
| Maternal alcohol use | 0.13 | 0.17 | -0.111 |
| AUDIT-positive | 0.24 | 0.25 | -0.035 |

*All standardized mean differences (SMD) ≤ 0.27, indicating no major selection bias on measured characteristics.*
